# Searching for mechanisms that matter in early septic acute kidney injury: an experimental study

**DOI:** 10.1186/cc10517

**Published:** 2011-10-26

**Authors:** Jan Benes, Jiri Chvojka, Roman Sykora, Jaroslav Radej, Ales Krouzecky, Ivan Novak, Martin Matejovic

**Affiliations:** 1 Department Of Anesthesia and Intensive Care, Charles University in Prague, Faculty of Medicine in Plzen, University Hospital Plzen, alej Svobody 80, 304 60, Czech Republic; 2 ICU, 1st Medical Dept., Charles University in Prague, Faculty of Medicine in Plzen, University Hospital Plzen, alej Svobody 80, 304 60, Czech Republic

## Abstract

**Introduction:**

In almost half of all sepsis patients, acute kidney injury (AKI) develops. However, the pathobiologic differences between sepsis patients with and without AKI are only poorly understood. We used a unique opportunity to examine dynamic inflammatory, renal hemodynamic, and microvascular changes in two clinically relevant large-animal models of sepsis. Our aim was to assess variability in renal responses to sepsis and to identify both hemodynamic and nonhemodynamic mechanisms discriminating individuals with AKI from those in whom AKI did not develop.

**Methods:**

Thirty-six pigs were anesthetized, mechanically ventilated, and instrumented. After a recovery period, progressive sepsis was induced either by peritonitis (*n *= 13) or by continuous intravenous infusion of live *Pseudomonas aeruginosa *(*n *= 15). Eight sham operated-on animals served as time-matched controls. All animals received standard intensive care unit (ICU) care, including goal-directed hemodynamic management. Before, and at 12, 18, and 22 hours of sepsis, systemic and renal (ultrasound flow probe) hemodynamics, renal cortex microcirculation (laser Doppler), inflammation (interleukin-6 (IL-6), tumor necrosis factor-alpha (TNF-α), oxidative stress (thiobarbituric acid reactive species (TBARS), nitrite/nitrate concentrations (NOx), and renal oxygen kinetics and energy metabolism were measured.

**Results:**

In 14 (50%) pigs, AKI developed (62% in peritonitis, 40% in bacteria infusion model). Fecal peritonitis resulted in hyperdynamic circulation, whereas continuous bacteria infusion was associated with normodynamic hemodynamics. Despite insults of equal magnitude, comparable systemic hemodynamic response, and uniform supportive treatment, only those pigs with AKI exhibited a progressive increase in renal vascular resistance. This intrarenal vasoconstriction occurred predominantly in the live-bacteria infusion model. In contrast to AKI-free animals, the development of septic AKI was preceded by early and remarkable inflammatory response (TNF-α, IL-6) and oxidative stress (TBARS).

**Conclusions:**

The observed variability in susceptibility to septic AKI in our models replicates that of human disease. Early abnormal host response accompanied by subsequent uncoupling between systemic and renal vascular resistance appear to be major determinants in the early phase of porcine septic AKI. Nonuniform and model-related renal hemodynamic responses that are unpredictable from systemic changes should be taken into consideration when evaluating hemodynamic therapeutic interventions in septic AKI.

## Introduction

The major importance of sepsis and acute kidney injury (AKI) as a clinical and public health issue has clearly been underscored by recent studies showing a steadily increasing incidence of both syndromes [1,2]. Although AKI is often a complex multifactorial syndrome, sepsis and septic shock prevail as its dominant causes [3]. The expanding population of patients with sepsis and AKI and the associated excess mortality provide a strong basis for further research aimed at establishing the pathomechanisms and addressing more rigorously all potentially modifiable factors to reduce this burden to patients and the health care system. Considering that AKI affects 40% to 50% of all sepsis patients [4-6], one of the fundamental, yet unresolved, questions is why AKI develops in some patients with sepsis, whereas the others are spared. Because both hemodynamic and inflammatory pathogenic pathways contribute to development of septic AKI (S-AKI), it is essential to understand hemodynamic and nonhemodynamic differences between subjects with S-AKI and those without AKI [7,8]. Although preliminary data in humans suggest that S-AKI is associated with systemic inflammation [9,10], limited ability to analyze exactly the renal pathophysiology in humans emphasizes the need for complex, dynamic, and clinically relevant animal studies [11]. Paradoxically, these animal models should replicate not only the complexity of human sepsis, but also the variability among animals closely mimicking that seen in clinical practice [11]. We took the unique opportunity to analyze dynamic inflammatory, renal hemodynamic, and microvascular changes in two clinically relevant large-animal models that replicate many of the biologic features of human septic shock, including the integration of standard day-to-day care resuscitative measures. Our aim was to assess variability in renal responses to sepsis and to identify both hemodynamic and nonhemodynamic mechanisms that distinguish individuals with AKI from AKI-free subjects. Because this modeling allowed us to generate septic animals both with and without S-AKI, the confounding effects of sepsis that are independent of AKI could be isolated and studied [12].

## Materials and methods

All experiments were performed in adherence to the National Institutes of Health *Guidelines on the Use of Laboratory Animals*, and their protocols were approved by the University Animal Care Committee. Thirty-six domestic pigs of either sex with a median body weight of 35 (32 to 40) kg were used. Eight time-matched sham-operated controls and 15 animals with sepsis induced by continuous infusion of live bacteria (*Pseudomonas aeruginosa*) were studied prospectively. In addition, we performed a secondary analysis of 13 septic animals that served as control groups in two randomized prospective studies [13,14]. In these animals, sepsis was induced by fecal peritonitis. These two studies addressed completely different questions, and the protocols did not interfere with each other.

### Anesthesia and surgical preparation

Anesthesia was induced with intravenous propofol (1 to 2 mg/kg) and ketamine (2 mg/kg). Animals were intubated and mechanically ventilated with tidal volumes of 8 to 10 ml/kg, with positive end-expiratory pressure of 0.6 kPa, and FiO_2 _of 0.4. Respiratory rate was adjusted to maintain normocapnia (arterial carbon dioxide tension, 4.0 to 5.0 kPa). During the surgical procedure, continuous infusions of fentanyl (10 to 15 μg/kg/h), thiopental (10 mg/kg/h), and pancuronium (4 to 6 mg/h) were administered. After surgical preparation, the infusion of thiopental and fentanyl was decreased to 5 mg/kg/h and 5 μg/kg/h, respectively, and maintained until the end of the experiment. Continuous infusion of Plasmalyte (Baxter Healthcare, Deerfield, IL, USA) or Ringerfundin solutions (Braun Melsungen Ag, Melsungen, Germany) were used as a fluid replacement in doses of 15 ml/kg/h during the surgery and reduced to 7 ml/kg/h thereafter. Normoglycemia (arterial blood glucose level, 4.5 to 7 mmol/L) was maintained throughout the whole experiment by using 20% glucose infusion as needed.

Before the surgical procedure, a fiberoptic arterial catheter was inserted into the femoral artery for continuous blood pressure measurement, intermittent double-indicator transpulmonary dilution (COLD Z-021; Pulsion Medical Systems GmbH, Munich, Germany), and blood sampling. Central venous and pulmonary artery catheters were introduced via jugular veins. Afterward, a midline laparotomy was performed, and a precalibrated ultrasound flowprobe (Transonic Systems, Ithaca, NY, USA) was placed around the left renal artery. Laser Doppler probe (PF 404, Suturable angled probe; Perimed, Jarfalla, Sweden) was placed directly over the renal cortex for cortical microcirculation assessment, and a double-lumen catheter was inserted into the left renal vein for renal venous pressure measurements and blood sampling. Peritoneal drainage was inserted before abdominal wall closure, and epicystostomy was performed under ultrasound control. A recovery period of 6 hours was provided before the baseline measurement.

### Measurements and calculations

At each time point (baseline, 12, 18, and 22 hours after induction of sepsis), the measurement of hemodynamics included cardiac output (CO), systemic vascular resistance (SVR), intrathoracic blood volume (ITBV), filling pressures of both ventricles (CVP, PAOP), renal artery blood flow (Q_ren_), renal venous pressure (RVP), and renal cortical microcirculation (LDF), as reported previously [13-15]. Renal vascular resistance was calculated according to the formula:

$$\text{RVR}=\left[\text{MAP }\left(\text{mm Hg}\right)-\text{RVP }\left(\text{mm Hg}\right)\right]∕{\text{Q}}_{\text{ren}}\left(\text{l}.\text{mi}{\text{n}}^{-\text{1}}\right).$$

Arterial, mixed venous, and renal venous blood samples were analyzed for pH, pO_2_, pCO_2_, and for hemoglobin oxygen saturation. Systemic oxygen delivery, systemic oxygen uptake, and renal oxygen delivery and oxygen uptake were derived from the appropriate blood gases and flow measurements.

Arterial and renal venous lactate and pyruvate concentrations were measured. Arterial blood samples were analyzed for plasma creatinine, leukocyte and platelet counts, tumor necrosis factor alpha (TNF-α), and interleukin 6 (IL-6). Oxidative and nitrosative stress was evaluated by measuring thiobarbituric acid reactive species (TBARS) and nitrate/nitrite (NOx) concentrations. To correct for dilution effects resulting from volume resuscitation, NOx, TBARS, ADMA, IL-6, and TNF-α levels were normalized for plasma protein content [13-15]. AKI was defined according to the AKIN criteria as an increase of more than 26.4 μmol/L or 150% in serum creatinine from baseline [16].

### Experimental protocols

The study consisted of three arms; intravenous live bacteria infusion (INFUSION, *n *= 15), fecal peritonitis (PERITONITIS, *n *= 13), and sham-operated control group (CONTROL, *n *= 8). In the infusion group, a continuous central venous infusion of *Pseudomonas aeruginosa *(strain O1 isolated from a patient with suppurative otitis, 1 × 10^9 ^colony-forming units/ml determined serial dilution and colony counts) was commenced after baseline data acquisition and maintained until the end of the study. The infusion rate was titrated to a clinical goal of moderate pulmonary hypertension (MPAP, 35 to 40 mm Hg). To avoid any variations in virulence, all pigs were challenged with bacteria from the same bacterial strain. In the peritonitis group, fecal peritonitis was induced by inoculating 0.5 g/kg of autologous feces incubated in 200 ml saline for 8 hours at 37°C through the drains into the abdomen.

In addition to crystalloid solution, 6% hydroxyethyl starch 130 kD/0.4 (Voluven 6%; Fresenius Kabi Deutschland GmbH, Bad Homburg, Germany) was infused to maintain normovolemia in a goal-directed fashion, guided by filling pressures response and ITBV measurement. Continuous IV noradrenaline was administered if mean arterial pressure (MAP) fell below 65 mm Hg and titrated to maintain MAP above 70 mm Hg. When the last set of data had been obtained, the animals were killed by potassium chloride injection under deep anesthesia, and section was performed.

### Statistical analysis

All values shown are median and interquartile range. The calculations were done by using SigmaStat software version 3.5 (Systat Software Inc., Erkrath, Germany). After exclusion of normality by using the Kolmogorov-Smirnov test, time-dependent changes within each group were tested by using the Friedman ANOVA on ranks and, subsequently, the Dunn test for multiple comparisons with the Bonferroni correction. Differences between the groups were analyzed by using the Kruskal-Wallis one-way analysis of variance on ranks or the Mann-Whitney rank sum test when only two sets of measurements were compared. A value of *P *< 0.05 was regarded as statistically significant.

## Results

### Characterization of sepsis models

All animals completed the whole protocol. Hemodynamic and oxygen-exchange parameters, inflammatory responses, oxidative stress, and other laboratory parameters are summarized in Table 1. Fecal peritonitis induced a hyperdynamic circulatory state with an increased cardiac output, whereas continuous bacteria infusion resulted in normodynamic circulation. Sepsis significantly decreased systemic vascular resistance in both groups, the effect being more pronounced in the Peritonitis group. Although pigs in the Infusion group received higher cumulative amount of fluids (469 (407 to 516) versus 387 (344 to 425) ml/kg in the Infusion and Peritonitis groups, respectively; *P *= 0.007), fluid resuscitation maintained ITBV without intergroup differences, suggesting comparable cardiac preload. Twelve (85%) animals in the Infusion group received noradrenaline infusion, whereas seven (54%) pigs required vasopressor support in the Peritonitis group to maintain MAP ≥ 70 mm Hg. The average dose of noradrenalin was higher in the Peritonitis group (1.47 (0.69 to 2.67) versus 0.25 (0.1 to 0.54) μg/kg/min; *P *= 0.012), but the time to start the vasopressor infusion was similar in both groups (1,277 (1,205 to 1,335) versus 1,035 (869 to 1,401) minutes in the Infusion and Peritonitis groups, respectively). In the Peritonitis group, the increased cardiac output resulted in a significant increase of systemic oxygen delivery, whereas systemic oxygen consumption did not change in any of the three groups over time. Sepsis induced acute lung injury in both sepsis groups, as documented by significant, progressive deterioration of the PaO_2_/FiO_2 _ratio. Both models of sepsis markedly increased plasma levels of TNF-α and IL-6, without statistically significant intergroup differences. These changes were accompanied by a remarkable increase of thiobarbituric acid reactive species (TBARS) levels, in particular in the peritonitis model, providing the evidence for oxidative stress.

**Table 1:** Characterization of sepsis models

	Baseline	12 hours	18 hours	22 hours
**Mean arterial pressure (mm Hg)**				
Control	95 [87-109]	86.5 [83-94]	80 [73-90]	84 [68-87]
Infusion	112 [103-122]	88 [79-100]^a^	82 [77-90]^a^	79 [74-88]^a^
Peritonitis	97 [92-104]	89 [73-99]	81 [69-97]^a^	76 [68-91]^a^
**Cardiac output (ml/kg)**				
Control	98 (90-105)	97 (85-100)	80 (76-87)	85 (77-98)
Infusion	90 (80-101)	98 (80-128)	98 (81-130)	106 (83-145)
Peritonitis	81 (67-97)	118 (97-148)^a^	129 (110-157)^ab^	152 (101-181)^ab^
**Systemic vascular resistance (dyne.s.cm^-5^)**				
Control	2,453 (1,838-2,700)	2,075 (1,935-2,299)	1,961 (1,872-2,169)	1,912 (1,648-2,262)
Infusion	2,457 (1,956-2,643)	1,562 (1,384-1,788)^a^	1,382 (1,116-1,698)^ab^	1,179 (873-1,692)^a^
Peritonitis	2,737 (2,003-2,858)	1,635 (1,102-1,978)^b^	1,165 (849-1,595)^ab^	853 (651-1,393)^ab^
**ITBVI (ml/kg)**				
Control	31 (28-33)	24 (21-34)	23 (21-30)	26 (23-28)
Infusion	27 (22-29)	25 (22-27)	24 (20-28)	23 (21-24)
Peritonitis	23 (22-26)	26 (22-31)	27 (23-29)	23 (19-34)
**DO_2 _systemic (ml/min/kg)**				
Control	11.9 (9.9-12.2)	11.1 (9.3-12.1)	9.2 (8.7-10.7)	9.6 (8.8-10.3)
Infusion	11.5 (9.6-12.5)	12 (9.2-15.5)^c^	12.2 (10.1-15)	11.9 (9.6-16.1)^c^
Peritonitis	11.4 (9-13.6)	16 (14.5-23.2)^b^	16.4 (15-21.9)^ab^	22.1 (13.4-24.5)^ab^
**VO_2 _systemic (ml/min/kg)**				
Control	5.6 (4.6-5.9)	5.5 (5.2-5.7)	4.8 (4.7-5.8)	3.3 (2.7-4.3)^a^
Infusion	4.5 (4.2-5.0)	5.2 (5-7.4)^a^	5.1 (4.7-6.9)	4.6 (3.5-6.2)
Peritonitis	4.8 (4.3-5.6)	5.9 (5.2-6.8)	5.6 (4.8-6.3)	7.2 (4.2-9.5)
**PaO_2_/FiO_2 _(mm Hg)**				
Control	489 (445-514)	399 (374-456)	412 (320-434)	432 (272-449)
Infusion	463 (348-502)	251 (164-352)^ab^	292 (191-350)	135 (66-205)^ab^
Peritonitis	456 (336-497)	340 (309-381)	173 (115-305)^b^	137 (80-286)^a^
**TNF-α (pg/g of protein)**				
Control	1 (1-5)	3 (1-5)	2 (1-6)	2 (1-5)
Infusion	2 (2-3)	5 (4-7)^a^	16 (10-23)^ab^	13 (7-25)^ab^
Peritonitis	1 (1-2)	7 (5-10)^ab^	13 (5-24)^ab^	18 (5-33)^ab^
**IL-6 (pg/g of protein)**				
Control	2 (1-2)	1 (1-1)^a^	1 (0-1)^a^	1 (1-2)
Infusion	2 (1-5)	7 (5-18)^b^	34 (27-101)^ab^	72 (23-175)^ab^
Peritonitis	3 (1-5)	29 (11-193)^ab^	126 (16-484)^ab^	203 (20-1,296)^ab^
**TBARS (nmol/g of protein)**				
Control	16 (14-18)	19 (16-28)^a^	24 (18-34)^a^	23 (16-26)^a^
Infusion	14 (13-15)^c^	22 (19-27)^c^	32 (23-39)^ac^	30 (27-46)^ac^
Peritonitis	18 (17-24)	52 (37-76)^ab^	84 (51-114)^ab^	70 (46-107)^ab^

Control, sham-operated group; DO_2 _systemic, systemic oxygen delivery; IL-6, interleukin 6; Infusion, sepsis induced by bacterial infusion; ITBV, intrathoracic blood volume; PaO_2_/FiO_2_, ratio between arterial oxygen tension and the inspired fraction; Peritonitis, peritonitis-induced group; TBARS, thiobarbituric acid-reactive species; TNF-α, tumour necrosis factor alpha; VO_2 _systemic, systemic oxygen uptake; ^a^significant difference within each group versus baseline (*P *< 0.05); ^b^significant difference between the Control group and any of the septic groups (*P *< 0.05); ^c^significant difference of the Infusion versus the Peritonitis group (*P *< 0.05).

### Characteristics of animals with and without S-AKI

Overall, S-AKI developed in 14 (50%) of 28 animals; 62% met criteria for AKI in the Peritonitis group, and 40%, in the Infusion group. Within the period of the experiment, 86% of these pigs reached AKIN stage I, and 14%, AKIN stage II. No statistically significant differences in any measured variables were found between AKI-free and AKI pigs at baseline. The gender distribution was homogeneous in both groups. Similarly, the total amount of infused *P. aeruginosa *was identical (24 (18 to 43) ml in the AKI-free versus 24 (16 to 35) ml in the AKI group). In the Peritonitis group, the exact quantification of bacterial load could not be assessed. Nevertheless, no statistically significant intergroup differences in systemic hemodynamic variables were seen during the course of the experiment (Table 2). In addition, neither total amount of fluids nor vasopressor requirement differed between AKI and AKI-free animals, suggesting comparable infectious insults. Nine of 14 (64%) pigs in the AKI group and 10 (71%) of 14 AKI-free animals were given noradrenaline infusion. Total dose of noradrenalin was 0.21 (0.01 to 0.42) versus 0.12 (0.05 to 0.22) mg/kg; *P *= 0.56 in the AKI and the AKI-free groups, respectively. Likewise, time to start the noradrenalin infusion was identical (935 (816 to 1,368) minutes for AKI versus 1,133 (900 to 1,420) minutes for AKI-free; *P *= 0.87).

**Table 2:** Characteristics of animals with and without S-AKI

	Baseline	12 hours	18 hours	22 hours
**Mean arterial pressure (mm Hg)**				
AKI	106 (97-115)	82 (72-99)^a^	82 (70-96)^a^	76 (62-88)^a^
Non-AKI	104 (90-114)	92 (80-100)	82 (76-91)^a^	78 (73-87)^a^
Control	95 (87-109)	87 (83-94)	80 (73-90)	84 (68-87)
**Cardiac output (ml/kg)**				
AKI	93 (68-103)	103 (94-139)	125 (97-157)^b^	107 (93-153)
Non-AKI	85 (79-97)	106 (76-137)	109 (82-129)^a^	145 (98-168)^ab^
Control	98 (90-105)	97 (85-100)	80 (76-87)	85 (77-98)
**Systemic vascular resistance (dyne.s.cm^-5^)**				
AKI	2,693 (1,912-2,826)	1,437 (846-1,840)^ab^	1,097 (722-1,333)^ab^	1,121 (809-1,663)^ab^
Non-AKI	2,477 (2,355-2,712)	1,676 (1,539-1,973)^a^	1,489 (1,282-1,699)^ab^	1,113 (819-1,668)^ab^
Control	2,453 (1,838-2,700)	2,075 (1,935-2,299)	1,961 (1,872-2,169)	1,912 (1,648-2,262)
**ITBVI (ml/kg)**				
AKI	23 (21-29)	25 (19-30)	27 (22-31)	21 (18-24)
Non-AKI	26 (23-29)	26 (23-30)	24 (21-26)	24 (22-29)
Control	31 (28-33)	24 (21-34)	23 (21-30)	26 (23-28)
**Fluid intake (ml/kg/h)**				
AKI	12 (9-14)	16 (15-17)^a^	18 (14-18)^a^	13 (9-16)
Non-AKI	13 (12-15)	18 (17-22)^a^	18 (17-22)	17 (13-22)
Control	13 (11-17)	16 (13-21)	13 (13-18)	13 (10-15)
**VO_2 _systemic (ml/min/kg)**				
AKI	4.8 (4.6-5.2)	6.0 (5.0-7.4)	5.6 (4.8-7.0)	5.8 (4.2-8.6)
Non-AKI	4.4 (4.1-5.1)	5.4 (4.9-6.2)	5.3 (4.6-6.1)	4.2 (3.5-7.3)
Control	5.6 (4.6-5.9)	5.5 (5.2-5.7)	4.8 (4.7-5.8)	3.3 (2.7-4.3)^a^
**Arterial base excess (mmol/L)**				
AKI	6 (5-8.5)	4 (-0.6-7.5)	2.7 (-8.6-6.5)	1.4 (-8.7-3.8)^ab^
Non-AKI	6.6 (4.7-11.8)	5.6 (4.7-8.5)	4.1 (2.7-6.7)	2.2 (1.1-7.7)^a^
Control	8.5 (6.9-12.6)	7.7 (6.7-8.3)	7.6 (5.1-11.2)	6.1 (5.1-7.3)
**Arterial lactate-to-pyruvate ratio**				
AKI	10 (5-18)	15 (10-23)	36 (19-72)^a^	38 (21-60)^a^
Non-AKI	10 (8-22)	12 (10-15)	22 (10-39)	20 (10-85)
Control	9 (8-26)	17 (12-29)	17 (11-75)	15 (10-37)
**NOx (μmol/g of protein)**				
AKI	0.8 (0.6-1)	1 (0.7-1.7)	1.4 (1-2)	1.3 (1-1.7)^a^
Non-AKI	0.8 (0.7-1)	0.8 (0.5-1.2)	0.9 (0.7-1.3)	1 (0.8-1.7)^a^
Control	1.1 (0.8-1.4)	0.6 (0.5-0.7)^a^	0.7 (0.6-0.9)	0.8 (0.6-1)

Control, sham-operated group; IL-6, interleukin 6; Infusion, sepsis induced by bacterial infusion; ITBV, intrathoracic blood volume; NO_x_, nitrite/nitrate concentration; PaO_2_/FiO_2_, ratio between arterial oxygen tension and the inspired fraction; Peritonitis, peritonitis-induced group, TBARS, thiobarbituric acid-reactive species; VO_2 _systemic, systemic oxygen uptake; ^a^significant difference within each group versus baseline (*P *< 0.05); ^b^significant difference between the Control group and any of the septic groups (*P *< 0.05).

**Figure 1:**
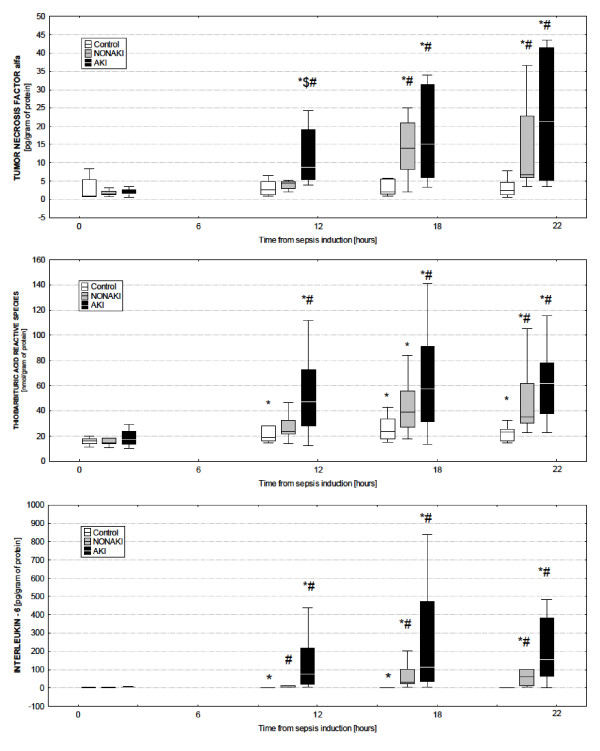
**Biomarkers of inflammatory response during the course of sepsis**. Plasma levels of inflammatory and oxidative stress markers (tumor necrosis factor α, upper panel; thiobarbituric acid-reactive species, middle panel; and IL-6, interleukin 6, lower panel) in control, non-AKI, and AKI animals. AKI, septic animals with acute kidney injury; control, sham operated; non-AKI, septic animals without acute kidney injury development. *Significant difference within each group versus baseline (*P *< 0.05); ^#^significant difference between the control group and any of the septic groups (*P *< 0.05); ^$^significant difference of the AKI versus the non-AKI group (*P *< 0.05).

Both groups had no changes in systemic oxygen consumption (Table 2). Significantly greater metabolic acidosis and higher arterial lactate/pyruvate ratio were noted in the animals that developed AKI over the ones that did not (Table 2). Despite comparable systemic hemodynamics and vasopressor support, only those animals with AKI had very early and remarkable increase in serum TNF-α and TBARS levels (Figure 1). Significant increase in these markers occurred later in the non-AKI group (Figure 1). Although a clear-cut tendency of plasma IL-6 levels was seen to increase more markedly in the AKI group, the intergroup differences did not reach statistical significance (Figure 1, lower panel).

### Renal effects of sepsis in pigs with and without AKI

**Figure 2:**
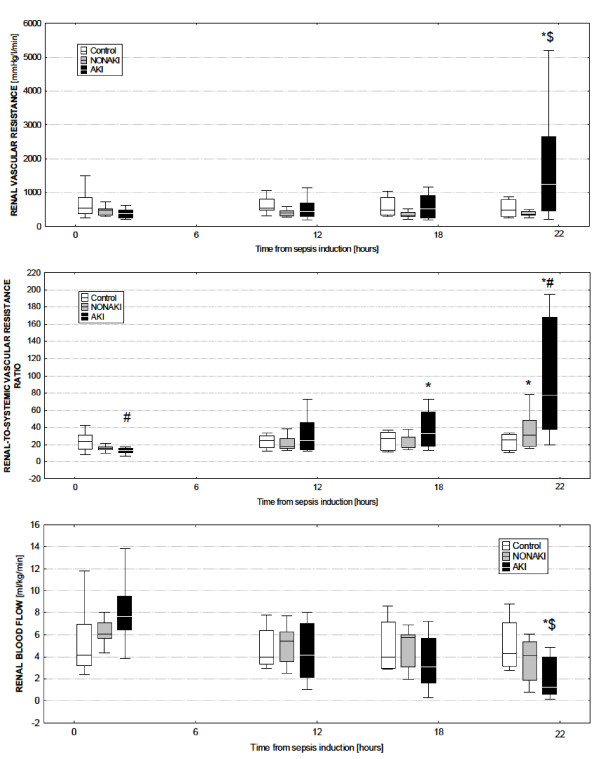
**Changes in renal hemodynamics during the course of sepsis**. Changes in renal vascular resistance (upper panel), renal-to-systemic vascular resistance ratio (middle panel), and renal blood flow (lower panel) in control, non-AKI, and AKI animals. AKI, septic animals with acute kidney injury; control, sham operated; non-AKI, septic animals without acute kidney injury developement; *significant difference within each group versus baseline (*P *< 0.05); ^#^significant difference between the control group and any of the septic groups (*P *< 0.05); $significant difference of the AKI versus the non-AKI group (*P *< 0.05).

Serum creatinine levels increased significantly in AKI animals by the end of the experiment, whereas no changes were observed in AKI-free and Control groups (Table 3). Urine output decreased markedly in AKI animals, whereas it transiently and significantly increased in AKI-free animals (Table 2). As opposed to subjects that remained AKI free, those in which AKI developed exhibited an increase in renal vascular resistance at the end of the experiment (Figure 2, upper panel). As compared with the Control group, the renal-to-systemic vascular resistances index more than doubled in the AKI groups (Figure 2, middle panel). This renal vasoconstriction, also apparent from the relative changes from baseline (RVR change, Table 3), was accompanied by a reduction in renal blood flow. No changes in renal blood flow occurred in AKI-free animals (Figure 2, lower panel). Despite maintained mean arterial pressure with noradrenaline above 70 mm Hg, renal perfusion pressure decreased significantly in both groups, partly as a result of gradually increased renal venous pressure (Table 3). Nevertheless, no significant intergroup differences occurred. Reduced renal blood flow observed in AKI animals was accompanied by decreased renal oxygen delivery and oxygen consumption at the end of the experiment, whereas no changes were detected in AKI-free pigs over time (Table 3). As compared with baseline values, the renal cortical microcirculatory blood flow decreased both in AKI-free and in the AKI group, with earlier deterioration observed in pigs with AKI (Table 3). Kidneys in AKI animals showed more marked metabolic stress, as evidenced by a significant development of renal venous metabolic acidosis and a tendency toward increased renal venous lactate/pyruvate ratio (Table 3).

**Table 3:** Renal effects of sepsis in pigs with and without acute kidney injury

	Baseline	12 hours	18 hours	22 hours
**Creatinine (μmol/L)**				
AKI	98 (88-105)	105 (91-134)	124 (89-164)	137 (130-176)^abc^
Non-AKI	94 (90-109)	96 (81-102)	95 (87-106)	101 (89-111)
Control	83 (75-96)	82 (72-100)	90 (76-101)	88 (77-99)
**Diuresis (ml/kg/h)**				
AKI	1.5 (1.3-2)	2.0 (1.6-2.8)	2.1 (0.9-4.7)	0.7 (0.1-1.2)^bc^
Non-AKI	1.9 (1.4-2.4)	4.2 (2.7-4.9)^a^	4.2 (2.2-6.4)^a^	1.5 (1.2-3.2)
Control	2.4 (1.8-3.2)	3.9 (2.3-6.6)	3.3 (2.5-5.7)	2.2 (1.8-3.2)
**Renal perfusion pressure (mm Hg)**				
AKI	91 (85-101)	70 (58-83)	64 (55-78)^a^	60 (53-73)^a^
Non-AKI	91 (76-97)	76 (64-83)	65 (57-71)^a^	59 (52-69)^a^
Control	86 (78-91)	70 (68-82)	67 (59-77)^a^	71 (51-77)^a^
**Renal venous pressure (mm Hg)**				
AKI	14 (12-15)	17 (15-19)	18 (18-19)^ab^	19 (18-21)^a^
Non-AKI	14 (11-15)	16 (15-17)	18 (15-20)^a^	20 (18-22)^a^
Control	11 (9-16)	14 (13-16)	14 (12-16)	17 (13-18)
**Change of renal vascular resistance from baseline (%)**				
AKI	100 (100-100)	114 (78-145)	111 (95-183)	242 (121-9,430)^abc^
Non-AKI	100 (100-100)	94 (75-110)	82 (58-113)	92 (69-112)
Control	100 (100-100)	112 (89-118)	101 (63-125)	81 (59-118)
**Renal VO_2 _(ml/min/kg)**				
AKI	0.2 (0.2-0.3)	0.2 (0.1-0.3)	0.2 (0.1-0.2)	0.1 (0-0.2)^a^
Non-AKI	0.2 (0.1-0.2)	0.2 (0.2-0.2)	0.2 (0.2-0.2)	0.2 (0.2-0.2)
Control	0.1 (0.1-0.2)	0.1 (0.1-0.2)	0.1 (0.1-0.2)	0.2 (0.1-0.2)
**Renal cortical microcirculation (laser Doppler flowmetry) (% of baseline value)**				
AKI	100 (100-100)	61 (43-80)^ab^	62 (45-97)^a^	48 (27-67)^a^
Non-AKI	100 (100-100)	70 (57-108)	70 (60-100)	61 (49-86)^a^
Control	100 (100-100)	97 (73-125)	70 (63-109)	73 (58-118)
**Base excess renal vein (mmol/L)**				
AKI	7.3 (6.1-8.5)	3.3 (0.1-6.7)^b^	2.9 (-5.6-6.7)	-1.9 (-11.8-3.3)^ab^
Non-AKI	8.9 (6.7-11)	6.2 (5.4-9.1)	4.9 (3.9-9.1)	4.1 (1.6-10.2)
Control	8.7 (7-13.7)	7.8 (6.9-9.7)	7.8 (5.3-9.6)	6.7 (4.8-7.4)
**Lactate/pyruvate ratio: renal vein**				
AKI	11 (7-17)	15 (12-21)	20 (9-43)	28 (18-43)
Non-AKI	10 (7-20)	7 (5-18)	13 (5-27)	10 (7-19)
Control	16 (9-21)	7 (6-20)	16 (7-32)	8 (6-17)

AKI, septic animals with acute kidney injury; Control, sham-operated group; DO_2 _systemic, systemic oxygen delivery; IL-6, interleukin 6; Infusion, sepsis induced by bacterial infusion; ITBV, intrathoracic blood volume; Non-AKI, septic animals without acute kidney injury development; PaO_2_/FiO_2_, ratio between arterial oxygen tension and the inspired fraction; Peritonitis, peritonitis-induced group; renal VO_2_, renal oxygen uptake; TBARS, thiobarbituric acid-reactive species; TNF-α, tumor necrosis factor alpha; VO_2 _systemic, systemic oxygen uptake; ^a^significant difference within each group versus baseline (*P *< 0.05); ^b^significant difference between the Control group and any of the septic groups (*P *< 0.05); ^c^significant difference of the AKI versus the Non-AKI group (*P *< 0.05).

### Renal effects of sepsis in pigs with AKI: comparison of sepsis models

**Figure 3:**
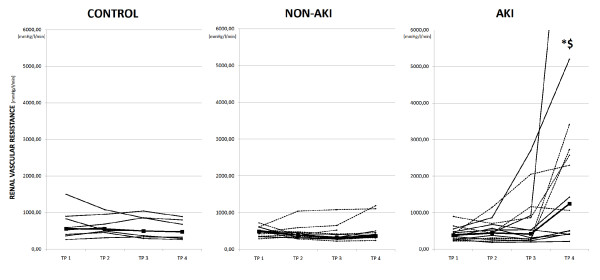
**Individual changes in renal vascular resistance in the AKI, non-AKI, and control groups**. Dashed line, infusion groups; solid line, Peritonitis and Control; bold line, median; TP 1 to 4, time points 1 to 4; AKI, septic animals with acute kidney injury; Control, sham-operated group; Infusion, sepsis induced by bacterial infusion; non-AKI, septic animals without acute kidney injury developement; Peritonitis, peritonitis-induced group; *significant difference within each group versus baseline (*P *< 0.05); ^$^significant difference of the AKI versus the non-AKI group (*P *< 0.05).

**Figure 4:**
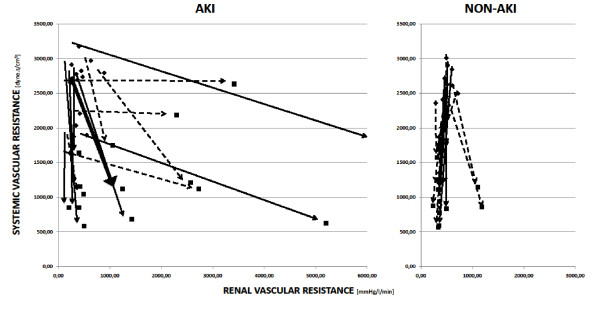
**Individual changes in renal and systemic vascular resistances in the AKI and non-AKI groups**. Lines indicate the individual connection between the baseline values of renal (x-axis) and systemic (y-axis) vascular resistances and the values measured after 22 hours of sespis. Arrow, the direction of change. Only AKI and non-AKI groups are displayed. Dashed line, infusion groups; solid line, peritonitis; bold line, median; AKI, septic animals with acute kidney injury; infusion, sepsis induced by bacterial infusion; non-AKI, septic animals without acute kidney injury developement; peritonitis, peritonitis-induced group.

Data comparing renal responses to different septic models are shown in Table 4 in the following order: bacteria infusion with AKI (AKI-INF; *n *= 6) and without AKI (NONAKI-INF; *n *= 9), and peritonitis with AKI (AKI-PERIT; *n *= 8) and without AKI (NONAKI-PERIT; *n *= 5). Individual responses are depicted in Figures 3 and 4. Increase in serum creatinine levels reached similar values in both groups at the end of the experiment (Table 4). No significant differences were noted in the amount of hydroxyethylstarch between any of the subgroups analyzed. Although renal vascular resistance increased gradually in the AKI group induced by live bacteria infusion, it remained unchanged in AKI animals challenged with peritonitis (Table 4 Figure 3). However, wide interindividual responses were observed even in the Peritonitis group (Figure 3). In addition, when compared with baseline values, the renal-to-systemic vascular resistances ratio increased in both groups of animals developing AKI, whereas it remained unchanged in AKI-free pigs in the Peritonitis group (Table 4). Individual relations between renal and systemic vascular resistances in the particular groups are shown in Figure 4. Renal blood flow and cortical microvascular perfusion decreased in both AKI models, without any intergroup differences. Despite apparent intrarenal vasoconstriction, only two (33%) animals in the AKI group induced by live bacteria infusion developed oliguria. By contrast, seven (88%) pigs became oliguric in the Peritonitis-induced AKI. No statistically significant differences in systemic inflammatory responses were found between pigs in both sepsis models with AKI, although those animals that developed AKI in the Peritonitis group had earlier and apparently greater increases of IL-6 levels. In addition, animals with AKI in the Peritonitis group had larger production of nitric oxide and reactive nitrogen species, as documented by significantly increased NOx and TBARS levels at 12 and 18 hours of sepsis (Table 4).

**Table 4:** Renal effects of sepsis in pigs with and without acute kidney injury: comparison of sepsis models

	Baseline	12 hours	18 hours	22 hours
**Creatinine (μmol/L)**				
AKI-INF	95 (84-114)	92 (88-148)	105 (85-168)	150 (134-180)^ab^
AKI-PERIT	98 (93-105)	107 (97-128)	129 (112-158)^c^	131 (129-166)^ac^
NONAKI-INF	106 (97-120)	100 (95-107)	102 (95-110)	109 (95-117)
NONAKI-PERIT	91 (84-91)	81 (78-96)	81 (77-91)	89 (84-94)
**Renal vascular resistance (mm Hg/L/min)**				
AKI-INF	359 (235-635)	373 (288-700)	576 (273-1,161)	2,433 (1061-2,727)^a^
AKI-PERIT	376 (302-459)	448 (280-637)	516 (236-821)	464 (404-1,425)
NONAKI-INF	471 (352-539)	388 (341-503)	415 (315-556)	355 (325-779)
NONAKI-PERIT	500 (431-541)	386 (320-407)	302 (273-340)^a^	383 (339-438)
**Renal-to-systemic vascular resistance ratio**				
AKI-INF	14 (11-17)	21 (13-32)	28 (17-58)	94 (49-170)^a^
AKI-PERIT	12 (10-14)	25 (14-59)	39 (20-59)^a^	38 (20-143)^a^
NONAKI-INF	15 (14-19)	18 (16-35)	23 (16-40)	30 (19-63)^a^
NONAKI-PERIT	16 (14-16)	18 (13-21)	16 (15-17)	24 (18-48)
**Renal flow (ml/min/kg)**				
AKI-INF	6 (6-11)	6 (3-8)	4 (2-7)	1 (1-2)^a^
AKI-PERIT	7 (5-8)	4 (3-5)	4 (2-6)	3 (1-4)^a^
NONAKI-INF	5 (4-7)	4 (3-5)	5 (3-5)	4 (2-5)
NONAKI-PERIT	6 (5-6)	7 (6-8)	7 (7-9)^a^	6 (4-6)
**Renal cortical microcirculation (laser Doppler flowmetry) (% of baseline value)**				
AKI-INF	100 (100-100)	70 (57-105)	67 (55-97)	35 (25-51)^a^
AKI-PERIT	100 (100-100)	59 (36-72)^a^	61 (41-85)	57 (32-75)^a^
NONAKI-INF	100 (100-100)	63 (53-111)	64 (52-101)	65 (47-92)
NONAKI-PERIT	100 (100-100)	88 (70-99)	75 (62-111)	59 (49-85)^a^
**Diuresis (ml/kg/h)**				
AKI-INF	1.7 (1-2.7)	2.7 (2.2-8)	4.8 (1.8-8.1)	1.1 (0.1-3.8)
AKI-PERIT	1.5 (1.3-1.9)	1.8 (1.5-2.3)	1.6 (0.9-2.1)	0.6 (0.2-0.9)^ac^
NONAKI-INF	2.2 (1.3-2.5)	4.5 (3.8-5.5)^a^	4.2 (1.7-5.4)	1.3 (1-2.3)
NONAKI-PERIT	1.6 (1.3-2.4)	2.3 (2-4.8)	3.9 (2.9-6.5)	1.7 (1.5-3.6)
**TNF-α (pg/g of protein)**				
AKI-INF	2 (2-3)	8 (6-19)^a^	14 (12-31)^a^	25 (20-42)^a^
AKI-PERIT	1 (1-3)	10 (5-16)	18 (5-30)^a^	18 (5-105)^a^
NONAKI-INF	2 (1-3)	4 (4-5)	18 (10-22)^a^	7 (6-15)^a^
NONAKI-PERIT	1 (1-1)	3 (2-7)	9 (5-15)	16 (6-30)^a^
**IL-6 (pg/g of protein)**				
AKI-INF	2 (1-5)	62 (5-104)	70 (33-130)^a^	155 (67-383)^a^
AKI-PERIT	3 (1-8)	118 (28-327)	246 (70-1,161)^a^	284 (68-3,981)^a^
NONAKI-INF	2 (2-5)	6 (5-10)	32 (27-51)^a^	61 (18-84)^a^
NONAKI-PERIT	3 (1-4)	12 (6-29)	19 (5-221)	33 (7-1,186)^a^
**NOx (μmol/g of protein)**				
AKI-INF	0.8 (0.6-1)	0.7 (0.6-0.7)	0.9 (0.7-1.4)	1.3 (0.9-1.5)
AKI-PERIT	0.8 (0.6-1.1)	1.6 (1.2-3)^ad^	1.6 (1.3-3.5)^a^	1.1 (1-2.1)
NONAKI-INF	0.9 (0.6-1.1)	0.5 (0.4-0.8)	0.7 (0.5-1)	0.8 (0.7-1.4)
NONAKI-PERIT	0.8 (0.7-0.9)	1.2 (1-1.5)	1.3 (0.8-1.7)	1.5 (1.3-1.7)^a^
**TBARS (pg/g of protein)**				
AKI-INF	14 (11-15)	26 (14-41)	31 (24-36)^a^	36 (27-61)^a^
AKI-PERIT	21 (18-25)^d^	68 (49-93)^ad^	90 (58-129)^ad^	75 (55-132)^a^
NONAKI-INF	14 (13-14)	22 (19-23)	32 (22-39)^a^	30 (27-40)^a^
NONAKI-PERIT	18 (16-21)	35 (32-48)	77 (44-91)^a^	62 (39-106)^a^

AKI, septic animals with acute kidney injury; Control, sham-operated group; DO_2 _systemic, systemic oxygen delivery; IL-6, IL-6, interleukin 6; interleukin 6; INF, sepsis induced by bacterial infusion; ITBV, intrathoracic blood volume; NONAKI, septic animals without acute kidney injury development; No_x_, nitrite/nitrate concentrations;PaO_2_/FiO_2_, ratio between arterial oxygen tension and the inspired fraction; PERIT, peritonitis-induced group; TBARS, thiobarbituric acid-reactive species; TNF-α, tumor necrosis factor alpha; VO_2 _systemic, systemic oxygen uptake; ^a^significant difference within each group versus baseline (*P *< 0.05); ^b^significant difference of the AKI-INF versus the NONAKI-INF group (*P *< 0.05); ^c^significant difference of the AKI-PERIT versus the NONAKI-PERIT group (*P *< 0.05); ^d^significant difference of the AKI-PERIT versus the AKI-INF group (*P *< 0.05).

## Discussion

We have developed two clinically relevant large-animal models allowing us to study the renal responses to sepsis. A unique feature of these models is variability in susceptibility to the development of S-AKI that closely replicates that of human disease. This heterogeneity allowed us to isolate and study factors discriminating AKI from non-AKI. Abnormal host response to sepsis, characterized by early systemic inflammation and oxidative stress coupled with subsequent renal vasoconstriction, was found to be a major discriminating factor associated with the initiation phase of S-AKI. Furthermore, our data suggest that in pigs developing S-AKI, renal circulation might not participate in sepsis-induced systemic vasodilatation. However, the intensity of renal vasoconstriction varies widely and appears to be model specific.

Ideally, animal models of sepsis-induced AKI should reproduce the mechanisms and consequences of human sepsis, including the complex supportive treatment. To increase the relevance of these models, they also should exhibit a heterogeneous response to sepsis that is comparable to that in humans [11]. In our study, 50% of animals developed S-AKI, a finding consistent with recent multicenter epidemiologic studies showing that in 40% to 50% of sepsis patients, concomitant AKI develops [4-6]. In addition, an early (that is, within 22 hours) development of S-AKI observed in our models also closely mimics a pattern seen typically in clinical studies [17]. The separation of animals into those with and without S-AKI according to the AKIN definition was associated with a number of significant physiological differences, adding validity and usefulness to this approach.

The most salient feature of the present study is the considerable variability in renal hemodynamic responses to sepsis despite a homogeneous and reproducible pattern of systemic hemodynamics and a uniform level of supportive treatment. This finding is somewhat contradictory to the results of a recent comprehensive review of the available experimental evidence, demonstrating that in the presence of preserved or increased cardiac output, renal blood flow is typically maintained or increased [18]. Similar predictive value was shown for calculated renal vascular resistance, although extraordinary heterogeneity exists [19]. We could consistently observe the link between well-maintained cardiac output and preserved renal blood flow only in those septic animals that remained AKI free. By contrast, in animals that developed AKI, sepsis is often accompanied by an uncoupling between systemic (reduced) and renal vascular resistance (increased), resulting in reduced renal blood flow despite maintained or even increased cardiac output. In contrast to all previous experiments, our study is the first to determine renal vascular resistance by using the directly measured renal venous pressure and to compare the differences between dynamic changes in renal vascular resistance in AKI-free and AKI-positive subjects. In this context, our results suggest that renal circulation might behave differently in pigs with S-AKI as opposed to S-AKI-free animals and that the renal circulatory response to sepsis cannot reliably be predicted from changes in systemic hemodynamics. Moreover, the divergent renal circulatory response observed in a majority of S-AKI animals supports the existence of the phenomenon of selective renal vasoconstriction, even in large-animal models with apparent sepsis-induced systemic vasodilatation [20]. Our observation is congruent with the clinical data of Lerolle *et al*. [21], who recently demonstrated that the increased Doppler resistive index, assessed at ICU admission as a surrogate measure of intrarenal vascular resistance, was significantly higher in septic shock patients in whom S-AKI subsequently developed. Conversely, our results markedly differ from a series of studies done by the Australian research group in a sheep sepsis model induced by continuous *Escherichia coli *infusion, in which S-AKI was uniformly associated with significant renal vasodilatation and increased renal artery blood flow [22-25]. Although the reasons for this discrepancy are not readily apparent, differences between species (sheep are ruminants) in regard to renal responses to sepsis, different bacterial insults (*E. coli *versus *Pseudomonas *vs. polymicrobial peritonitis) and the use of conscious versus anesthetized animals can be taken into consideration [18,26].

Based on the data presented here, we cannot unambiguously determine the cause of elevated renal vascular resistance. Recently, we observed gradually increased renal venous pressure with an early reduction in renal cortex microvascular perfusion during progressive porcine sepsis, each potentially contributing to the increase in renal resistance [15]. Although both renal venous congestion and deterioration in cortex microcirculation developed in this study, none of these variables differed from those in AKI-free animals. Renal interstitial tissue edema, more pronounced microvascular blood-flow derangement in deeper cortex layers and medulla not detected by our approach, and altered renal vasculature might have been factors implicated in the increased flow resistance in the S-AKI group. Whatever the mechanisms accounting for the increased renal resistance, the design of the present study does not allow one to determine a causative link between intrarenal vasoconstriction and kidney injury nor to clarify its relative contribution to S-AKI. However, reduced renal oxygen consumption, combined with signs of metabolic stress, as evidenced by increased renal venous base deficit implies an impairment of normal aerobic respiration. Because we did not calculate renal oxygen consumption per sodium reabsorbed, we can only speculate to what extent the oxygen consumption was affected by perturbed mitochondrial function or altered efficiency for tubular sodium transport. Nevertheless, considering the unchanged renal lactate clearance and oxygen extraction ratio (data not shown), it is plausible to speculate that the reduction in renal oxygen consumption represents decreasing oxygen demand rather than exhaustion of renal oxidative metabolism.

The onset of AKI was preceded by early and remarkable inflammatory response and oxidative stress. Despite comparable septic insult and indistinguishable systemic hemodynamic response, only those pigs that developed AKI had a very early increase in the plasma levels of TNF-α and TBARS. A similar pattern, albeit not statistically significant, was observed in the time-dependent changes of plasma IL-6 levels. These findings corroborate emerging evidence from laboratory and clinical studies suggesting that AKI in sepsis has a prominent inflammatory component in both the initiation and the extension phases of the kidney injury [12,27,28]. Several large cohorts of critically ill patients demonstrated that IL-6 could be a robust predictor of AKI [9]. In keeping with our findings, Murugan *et al*. [10] recently demonstrated different immune response to pulmonary infection in patients with AKI as opposed to patients without AKI. Patients admitted with pneumonia and who presented with or developed concomitant S-AKI had higher plasma levels of IL-6 and TNF-α than did non-AKI patients. Although our study is not the first to assess the association between inflammation and the susceptibility to S-AKI, it extends the clinical observations by demonstrating the temporal relation between early, exaggerated inflammatory and oxidative stress response and AKI, thus supporting the cause-effect interplay between AKI and abnormal immune response.

Another major feature of this study is the comparison of renal responses in two different sepsis models. To our knowledge, we are the first to demonstrate renal physiology in two different large-animal models of sepsis. First, they displayed differences with respect to the renal vascular response to sepsis. Although the ratio of renal to systemic vascular resistance increased in both groups when compared with baseline values, the increase in renal vascular resistance was more remarkable in pigs challenged with intravenous bacteria infusion. However, as shown in Figures 3 and 4, the individual responses were heterogeneous. The exact mechanism(s) responsible for different renal vascular responses cannot be inferred from our data. The preservation of urine output in the Infusion group was likely caused by an increase in the hydrostatic pressure of the glomerular capillaries, indicating that infusion of live bacteria elicited more vasoconstriction in the efferent than in the afferent arteriole of the glomerulus, which is in sharp contrast to results observed in a sheep model by Langenberg *et al*. [23]. Different renal vascular responses might be attributable to different immune responses. Only in those pigs in the Peritonitis group that developed S-AKI, was a significant, albeit transient, increase present in the production of nitric oxide and oxidative stress, as revealed by changes in plasma levels of NOx and TBARS, respectively. These results could explain the relatively preserved renal vascular resistance in the Peritonitis-induced AKI model. In addition, the inflammatory response evaluated by plasma IL-6 levels appeared to be more pronounced in this model than that elicited by bacteria infusion. Of note, S-AKI developed more frequently in the Peritonitis group (62%) as compared with the Infusion group (40%). Collectively, these data support a prominent role of abnormal host response to the infection in the development of S-AKI and suggest that renal vasoconstriction might not necessarily be a prerequisite for AKI to develop, at least in the Peritonitis model [7]. Although inconclusive data exist regarding the association of AKI and primary infectious source [5,17,29], our data admit the dependence of physiological renal responses on the causative agent(s) and infectious source. Certainly, knowing histopathologic findings in all particular subgroups would allow us to put the data into a more comprehensive picture. Because renal tissue samples taken from our animals are scheduled for detailed genomic and proteomic analyses, we were unable to provide morphologic data at this stage.

## Conclusions

In conclusion, we aimed to assess variability in renal physiology and to study both hemodynamic and nonhemodynamic factors discriminating AKI from non-AKI in two different large-animal models that closely resemble human sepsis. The lessons that can be drawn from these models are that (a) they exhibited marked heterogeneity in susceptibility to the development of septic AKI that is comparable to that in humans; and (b) the differences between those animals that developed AKI and those that did not are noteworthy from both the clinical and research perspectives. First, although sepsis appears to be a uniform clinical entity, much variation can be seen in the renal responses. This variability has to be taken into account when effects of therapeutic interventions are to be assessed. Second, renal circulatory response to sepsis cannot reliably be predicted simply from changes in systemic hemodynamics. Renal blood flow can exhibit significant variability, even though cardiac output is maintained or increased. Third, the development of S-AKI is often accompanied by an uncoupling between systemic and renal vascular resistance, suggesting that renal circulation does not participate in sepsis-induced systemic vasodilatation. Fourth, early abnormal host response seems to be a major pathobiologic factor associated with the development of S-AKI. Fifth, the intensity of immune and renal hemodynamic responses appears to be model specific. The different renal responses of these models to sepsis could allow future studies to tease apart the underlying pathophysiology of S-AKI.

## Key messages

• Although sepsis appears to be a uniform clinical entity, much variation can occur in the renal hemodynamic and functional responses. This variability has to be taken into account when effects of therapeutic interventions are to be assessed.

• Early abnormal host immune response appears to be the major pathobiologic factor predicting the development of porcine septic AKI.

• Renal circulatory response to sepsis in animals developing AKI cannot reliably be predicted from changes in systemic hemodynamics.

• Development of septic AKI is often accompanied by an uncoupling between systemic and renal vascular resistance, suggesting that renal circulation does not participate in sepsis-induced systemic vasodilatation.

## Abbreviations

AKI: acute kidney injury; AKI-free/non-AKI: without acute kidney injury; CO: cardiac output; CVP: central venous pressure; IL-6: interleukin 6; ITBV: intrathoracic blood volume; LDF: renal cortical microcirculation measured with laser-Doppler probe; MAP: mean arterial pressure; MPAP: mean pulmonary arterial pressure; NOx: nitrite/nitrate concentrations; PAOP: pulmonary artery occlusion pressure; Qren: renal artery blood flow; RVP: renal venous pressure; RVR: renal vascular resistance; S-AKI: sepsis-associated acute kidney injury; SVR: systemic vascular resistance; TBARS: thiobarbituric acid-reactive species; TNF-α: tumor necrosis factor alpha.

## Competing interests

The authors declare that they have no competing interests.

## Authors' contributions

MM obtained research funding, conceived the study, designed the study protocol, supervised and coordinated the study, and finalized the manuscript. JB carried out the experiments, statistical analysis, and drafted the article. JC and RS performed the experiments and data acquisistion. AK, JR, and IN participated in the design of the study, contributed to the interpretation of the results, and helped to draft the manuscript. All authors read and approved the final manuscript for publication.

## Authors' information

The study was performed in the animal research laboratory of the 1^st ^Medical Department at Charles University Medical School.

The study was presented in part at the 31^st ^International Symposium on Intensive Care and Emergency Medicine, Brussels, March 2011.
